# Inhibition of Platelet Adhesion from Surface Modified Polyurethane Membranes

**DOI:** 10.26717/BJSTR.2020.32.005247

**Published:** 2020-12-08

**Authors:** Shih-Feng Chou, Brandon A Caltrider, Ali Azghani, Pierre F Neuenschwander

**Affiliations:** 1 Department of Mechanical Engineering, The University of Texas at Tyler, USA; 2 Department of Biology, The University of Texas at Tyler, USA; 3 School of Medical Biological Sciences, The University of Texas Health Science Center at Tyler, USA

**Keywords:** Platelets, Activation and Adhesion, Coronary Thrombosis, Blood Clotting, Polyurethane, Surface Properties

## Abstract

Coronary thrombosis is one of the leading causes of mortality and morbidity in cardiovascular diseases, and patients who received vascular stent treatments are likely to suffer from restenosis due to tissue damage from stenting procedures (extrinsic pathway) and/or presence of unregulated factor XII (intrinsic pathway). Regardless of the pathway, coagulation factors and exposed collagen activate the G-protein-coupled receptors located at the plasma membrane of the resting platelets resulting in the change of their shapes with protrusions of filopodia and lamellipodia for surface adhesion. In this mini review, we discussed the mechanisms involved in platelet activation, adhesion, and aggregation. More importantly, we reviewed the use of polyurethane membranes with modified surface functional groups to down-regulate platelet adhesion and aggregation activities. Polyurethane membranes with hydrophilic and negatively charged surface properties showed a reduced αIIb-β3 signaling from the activated platelets, resulting in the decrease of platelet adhesion and aggregation. The use of polyurethane membranes with modified surface properties as coatings on vascular stents provides an engineering approach to mitigate blood clotting associated with restenosis.

## Introduction

Cardiovascular diseases account for 31% of all deaths (17.9 million people) worldwide according to the World Health Organization (WHO) [1]. One of the major cardiovascular diseases is coronary thrombosis, and it is associated with the narrowing and/or blockage of coronary arteries that prevent the circulation of blood to the heart tissue resulting in heart attacks and/or heart failures. Development of coronary thrombosis starts with the narrowing of the arteries due to the formation and growth of plaques, consisting of fatty products of cholesterols, calcium, and cellular wastes, inside the vessels wall. When a plaque ruptures, platelets are recruited and accumulated onto the surface of the plaque in order to stop vascular bleeding. The repeating process of plaque rupture and platelet accumulation inside the coronary arteries leads to coronary thrombosis [2]. The current therapeutic methods for this life-threatening cardiovascular disease include balloon angioplasty, vascular stenting, and vascular bypass grafts. However, restenosis, the reoccurrence of narrowed blood vessels associated with vascular tissue damage during angioplasty, can often promote thrombus formation within the first 24 hours that may lead to a second acute heart attack [3]. Placement of vascular stents, including the traditional bare metal stents and the recently developed drug-eluting stents, may cause in-stent restenosis associated with vascular tissue damage during implantation. Furthermore, vascular stents and vascular bypass grafts are considered as foreign objects to the body, which may trigger uncontrollable immune responses that facilitate coagulation. These unregulated enzymatic activities can be categorized into intrinsic (contact) and extrinsic (trauma) pathways that ultimately converge at the generation of factor X (FX) [4]. The generation of activated factor X (FXa) is then catalyzed by activated factor VIII (FVIIIa) and activated factor VII (FVIIa) through the intrinsic and extrinsic pathways, respectively [5]. Local blood clots are formed as FXa interacts with its cofactor FVa resulting in the production of prothrombinase complexes that generate thrombin (FIIa), which promotes the polymerization and deposition of insoluble fibrins. Both the intrinsic and the extrinsic pathways provide signaling proteins that activate platelets to up-regulate their activities in adhesion and aggregation.

In our previous work [4], we have provided a perspective on the applications of engineering membranes, including drug-eluting materials and surface modified polymeric and/or non-polymeric coatings on medical implants to improve hemocompatibility. In particular, our original research work indicated that polyurethane membranes slowed down blood coagulation by 1000-fold as compared to the glass control surfaces [6]. As a continuous effort to investigate the hemocompatibility of polymeric membranes, while providing an overview of current methods performed on the in vitro platelet adhesion assays, we review research works done in the field with modified polyurethane membranes. In this article, we describe the mechanisms involving platelet activation through intrinsic and extrinsic pathways of coagulation followed by narrating how platelets adhere to a surface while recruiting and aggregating more platelets through a positive feedback loop to clot the blood and stop blood loss. This mini review provides research works in the area of polyurethane membranes and its modified surfaces as potential substrates in preventing platelet activation, adhesion and aggregation. The applications of a thin polyurethane coating on the surface of implantable medical devices, such as a vascular stent, provide an engineering approach to safely down-regulate coagulation factors that activate platelets to adhere and aggregate into a blood clot.

## Platelet Activation and Adhesion

Platelets play a primary role in thrombosis and hemostasis due to their ability to self-activate in response to signaling cues. Platelet exposure to collagen and thrombin in an injured vessel due to extrinsic causes results in the activation of platelets. Through the intrinsic pathway; however, factors such as surface charges, hydrophobicity, and topography of a contact surface may activate platelets and promote their adhesion, aggregation, and activation [7,8]. In particular, unregulated factor XII (FXII), which is known to bind to collagen and initiate the coagulation process, promotes platelet activation. Studies have shown that the FXII-dependent procoagulant capacity of collagen increased significantly due to the release of platelet-derived activators such as linker for activation of T cells (LAT) and phospholipase Cγ2 PLCγ2 [9]. This finding is further supported by the role of integrin αIIb-β3 signaling from activated platelets in promoting FXII activation [10]. Irrespective of the method of activation (e.g., extrinsic or intrinsic causes), these signaling pathways alter platelets morphology and behavior where platelets spread more, adhere better, and overexpress filopodia and lamellipodia for further amplification of the signals [11].

**Figure 1:**
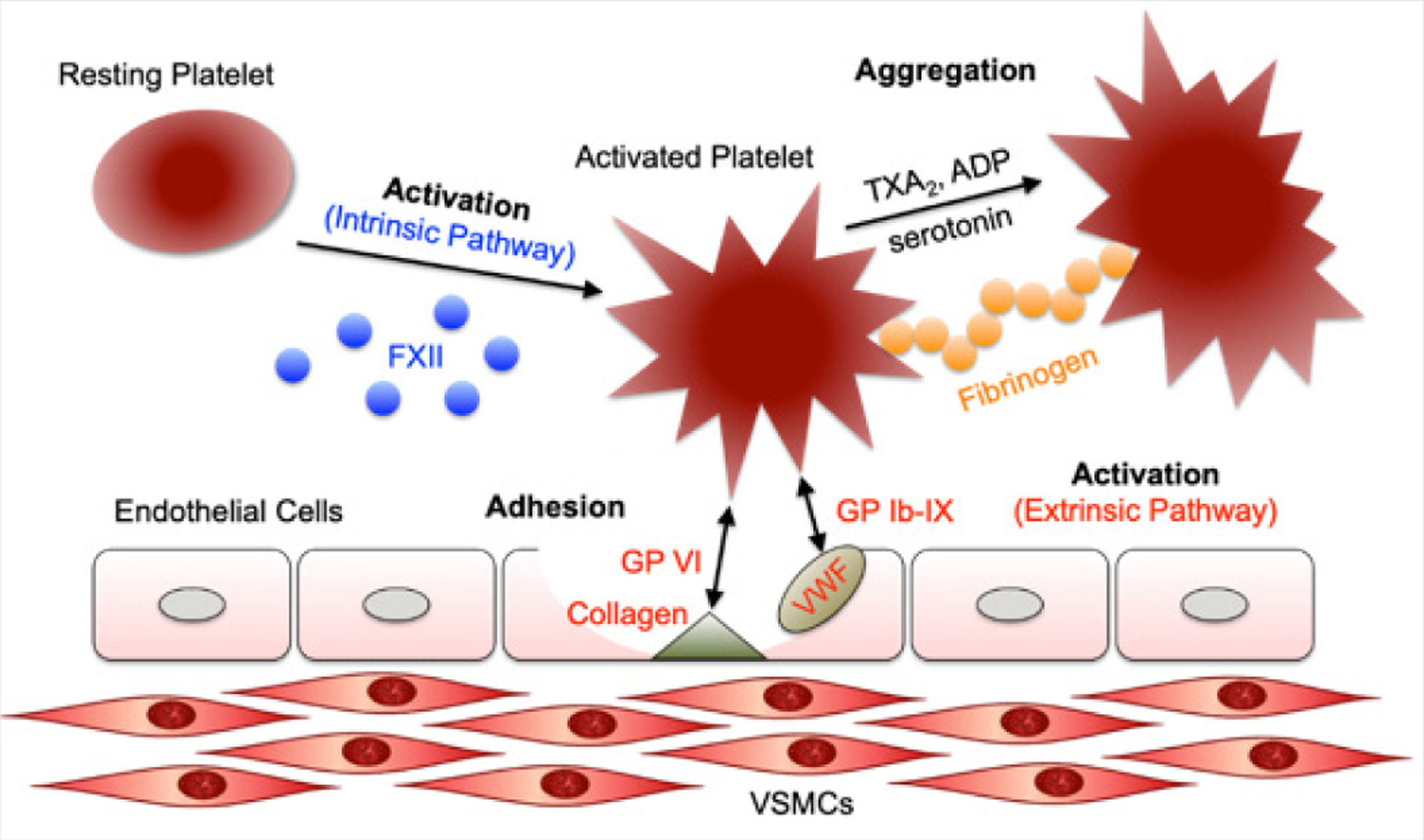
A schematic illustration on the mechanisms of platelet activation via intrinsic and/or extrinsic causes followed by platelet adhesion to a surface and the sequential signaling events in aggregation. Figure is redrawn from [12].

The mechanisms of platelet activation and the subsequent adhesion and aggregation processes are illustrated in Figure 1 [12]. As indicated, various adhesive Glycoprotein (GP) complexes on or in the platelets’ plasma membrane interact with extracellular proteins such as von Willebrand factor (GP Ib-IX) and collagen (GP VI) [13,14]. In addition, soluble platelet agonists such as thrombin, serotonin, Adenosine Diphosphate (ADP), and thromboxane A2 (TXA2) are released from the damaged cells as well as the activated platelets to recruit the circulating platelets via various G-Protein-Coupled Receptors (GPCRs) [15]. Thrombin activates platelets through Protease-Activated Receptors (PARs) to stimulate the approaching platelets [16]. Thrombin also binds with GP Ib-IX to activate platelets into low-dose thrombin. Serotonin binds with platelet Gq-coupled receptor, 5HT2A [15]. ADP, reserved in the granules of platelets, is the metabolic byproduct of cellular activities, and it activates the resting platelet through purinergic receptor P2Y12 [17]. TXA2 activates platelets through the TXA2/prostaglandin H2 receptor (TP) that couples with the GPCRs [18]. Other factors, such as calcium concentration in blood, regulate the physiologic function of platelets since the increase in calcium concentration promotes fibrinogen receptor activation [19].

Platelet adhesion and activation due to restenosis is of particular concern, especially for the design of engineering surfaces that inhibit blood clotting from platelet aggregation. For example, it has been shown that the negatively charged surfaces not only down-regulate the conversion of the adsorbed fibrinogen to fibrin [7], which impedes the coagulation process, but also reduce platelet aggregation through mechanisms of passive agglutination (platelets are negatively charged) and integrin αIIbβ3-mediated aggregation [20]. In addition, increasing surface hydrophobicity significantly increases fibrinogen adsorption, which promotes platelet adhesion [21]. However, hydrophobic surfaces that exhibit contact angles greater than 120 degrees show better blood compatibility and less potential for platelet adhesion and activation [22]. Finally, a 2.5-fold reduction on platelet aggregation suggests the effects of surface topography (i.e., from 700 nm to 400 nm) on bulk platelet activation [23]. In general, platelet activation and adhesion through intrinsic pathway of surface contacts of foreign objects are related to various cellular mechanisms on the activation of protein receptors as well as surface physicochemical properties of the implantable devices (e.g., vascular stents).

## Engineering Surfaces That Prevent Platelet Adhesion

Inhibition of pathologic platelet activation has been under intensive research efforts. Providing an engineering solution to prevent platelet adhesion and subsequent activation by implantable devices, such as vascular stents, is one such effort. Even though drugs such as aspirin are a potential preventive solution to restenosis, platelet aggregation on the other hand is a natural process that is necessary to maintain proper functioning of the body, especially when injury occurs [24]. To locally down-regulate platelet activation and adhesion ability while still being able to maintain its function in coagulation process, surface modifications of the implantable devices become an ideal engineering solution. This approach leads to the investigation of the platelet compatibility on Polyethylene Glycol–Polyurethane (PU-PEG) coatings. Implantable devices in contact with blood in current implementations have short lifespans predominately due to blood coagulation. Studies involving polymer coatings with modified surface functional groups have been conducted in an effort to provide a permanent solution for a hemocompatible device. A recent in vitro study indicated that the Polyethylene Glycol (PEG) modified polyurethane (PU) surfaces with different terminal groups (−OH, −NH_2_, and −SO_3_) prevents platelet activation and adhesion [25].

Results showed 1.3-fold and 1.7-fold increases of free calcium concentration in the PEG-modified PU surfaced terminated with hydroxyl and amine functional groups, respectively, as compared to the PEG-modified PU control groups after 20 minutes of incubation. The higher free calcium concentration in the PU surfaces terminated with amine functional groups was due to the ionic interactions between the amine and the platelet membrane. In addition, the sulfonate functionalized PEG-modified PU surfaces showed a 0.4-fold decrease in free calcium concentration as compared to the PU control groups, suggesting the dependence of platelet activation on surface functional groups of polymer coatings. In vitro platelet adhesion assays were performed on these surfaces, and the PEG-modified PU surfaces showed 47% less platelet adhesion than the blank PU controls after 10 minutes of incubation. Platelet adhesions decreased to 27%, 21%, and 12% on the PEG-modified PU surfaces terminated with hydroxyl, amine, and sulfonate functional groups, respectively. These findings demonstrate the effects of surface functional groups on the inhibition of platelet activation and adhesion.

In addition to the surface chemical nature and ionic charges, surface wettability of PU coatings can be used as an effective surface modifier [6]. In a study, soybean-derived Phosphatidylcholine (PC) modified PU coatings were prepared by dipping PU coatings in PC containing PU solutions followed by solvent evaporation [26]. The resulting PC-modified PU coatings exhibited an increase in surface wettability indicated by contact angle measurements. The PC-modified PU surfaces consisted of phosphorylcholine end groups on the coating surface, mimicking the physiologic endothelial membrane structure that significantly reduced the adsorption of plasma-derived proteins, such as fibrinogen, fibronectin, or von Willebrand factor. Enzyme-Linked Immunosorbent Assays (ELISA) compared the surface absorptions of fibrinogen in platelet poor plasma on the PC-modified PU to that of PU alone. The data showed a greater than 3-fold reduction in the amount of adsorbed fibrinogen on the PC-modified PU groups than the PU controls. Furthermore, hemocompatibility analysis under dynamic shear stress testing conditions using a platelet analyzer suggested that more than 70% of platelets were maintained in the blood samples after contacting the PC-modified PU surfaces.

Effects of polymer swelling ability have a direct influence on the physical cues of platelet adhesion. Different substituents at the N-position of poly(*N*-alkyl acrylamide) coatings were dip-coated onto PU substrates to investigate the swelling ability (hydrophilicity) of the coatings on the platelet adhesion [27]. These coatings, irrespective to the number and length of alkyl substituents at the N-position that attributed to varying swelling degree, exhibited little to negligible platelet adhesion due to the bioinertness of poly(*N*-alkyl acrylamides) that repelled proteins and endothelial cells. Furthermore, crosslinking of the poly(*N-*alkyl acrylamide) reduced the swelling ability and improved the mechanical properties of the coating under shear stress environment without affecting the thrombogenic properties of the surfaces by preventing platelet adhesion.

Surface modifications of the PU membranes with a small molecule antiplatelet drug, dipyridamole, demonstrated the abiity to reduce platelet adhesion. In a study, dipyridamole was used in two variations on the surface modification of the PU membranes [28]. The two groups included linking dipyridamole directly to PU membranes and linking dipyridamole to PU membranes with a short hydrophilic spacer chain. After 15, 30, and 60 minutes of incubation in human platelet rich plasma, platelet adhesion density of the dipyridamole-linked PU membranes decreased 72%, 35%, and 52% as compared to the PU controls, respectively. In addition, the platelet adhesion density from the dipyridamole-linked PU membranes with hydrophilic spacers decreased 63%, 28%, and 18% as compared to the PU controls, respectively. Interestingly, at short incubation time (e.g., 15 min), PU membranes grafted with only the hydrophilic spacers increased the platelet adhesion density by 130%. This trend was further decreased to 53% at 30 minutes and 27% at 60 minutes, suggesting that hydrophilic surfaces were more hemocompatible perhaps due to the mechanisms involved in platelet spreading and aggregation.

Incorporating Polyethylenimine (PEI) in PU membranes enhances the surface grafting sites of primary amine (−NH_2_). Tethering of heparin and phosphorylcholine (PC) groups on the surface of PEI-PU membranes inhibited platelet adhesion [29]. Water uptake tests demonstrated that blank PEI-PU membranes, heparin-tethered PU membranes, and PC-grafted PU membranes were 23.6×, 13.6×, and 15.9× more hydrophilic than the PU control groups, respectively. The low water contact angles (35°~40°) of these modified surfaces supported the water uptake data and showed that the surfaces of these membranes were hydrophilic. Static platelet adhesion assays using platelet rich plasma on PU control groups showed platelet aggregates, where the adhered platelets exhibited long pseudopods, which is evidence of platelet activation to spread over the surfaces. Platelet adhesion on PEI-PU membranes were reduced by 48%, whereas both heparin-tethered PU membranes and PC-grafted PU membranes demonstrated 1000-fold decreases in platelet adhesion.

Taken together and summarized in Table 1 [25–29], the surface modified PU membranes demonstrate the promising ability in inhibiting platelet activation and adhesion. These surfaces are either hydrophilic or swellable, which repel the proteins deposition during the signaling events of platelet adhesion and aggregation. In addition, surface modifications of PU membranes allow grafting of antiplatelet molecules to prevent the deposition of plates on the coating surfaces. These techniques provide a much-improved life span on the implantable medical devices, such as vascular stents.

## Conclusion and Future Directions

Platelets play a fundamental role in clot formation and preventing hemorrhage, a necessary and protective function in hemostasis. Thrombosis, however, is a pathologic state when unwanted blood clots are formed inside the arteries. Thrombosis may occur through the extrinsic (trauma) or intrinsic (contact) pathways, and both pathways converge into the formation of thrombin that activates the polymerization and deposition of fibrin. Each pathway provides important signaling factors to activate platelets via the G-protein-coupled receptors located at the plasma membrane. Once activated, filopodia and lamellipodia protrusions provide platelets with adhesion ability to the surface. Crosstalks between the activated platelets enable the aggregation of platelets into a blood clot.

Several polyurethane membranes with modified surface properties, either by promoting the surface wettability or enhancing the surface negative charge, have shown promising results in preventing platelet adhesion or aggregation. These types of thin coatings may be used on implantable medical devices (i.e., vascular stents) to improve their therapeutic functions in the body by reducing the chances of having a restenosis. The future of polymer coatings may facilitate the development of multi-functional membranes, such as a composite, to provide active, as well as passive, protections to the stents. In addition, surface grafting of anti-clotting functional groups tailored to patients’ needs is also a promising research topic in anticoagulant polymer membranes.

## Figures and Tables

**Table 1:** Summary of thrombogenicity of modified Polyurethane (PU) surfaces.

PU Modifications	Assays	Results	Mechanisms	[Ref]
PEG-modified PU membranes with −OH, −NH2, and −SO3 terminal groups	Static: fluorescence measurement of cytoplasmic free calcium.	27% reduction of platelet adhesion on PEG-PU (−OH) groups. 21% reduction of platelet adhesion on PEG-PU (−NH_2_) groups. 12% reduction of platelet adhesion on PEG-PU (−SO_3_) groups.	Inoic interactions between the surface functional end groups and platlet membrane.	[25]
Phosphatidylcholine (PC) modified PU membranes	Static: ELISA assays on fibrinogen adsorption; SEM. Dynamic: platelet flow analyzer.	Static: > 3-fold reduction of the fibrinogen adsorption on PC-modified PU membranes. Dynamic: > 70% of platelets remained in blood samples after contacting PC-modified PU membranes.	Mimicking of the physiologic endothelial membrane structure to reduce the adsorption of plasma-derived proteins.	[26]
Poly(*N*-alkyl acrylamide) modified PU membranes	Dynamic: flow chamber	Negligible platelet adhesion irrespective of varying swelling ratios and crosslinking of the membranes.	Bioinertness of poly(N-alkyl acrylamides) and hydrophilic surface properties that repels proteins.	[27]
Dipyridamole grafted PU membranes	Static: microscopic platelet counting assays Dynamic: platelet perfusion chamber	Static: 52% and 18% reduction on platelet adhesion using dipyridamole grafted PU membranes and dipyridamole grafted PU membranes with hydrophilic spacers, respectively, after 60 min of incubation. Dynamic: 40% and 30% of the platelets were immobilized on the dipyridamole grafted PU membranes and dipyridamole grafted PU membranes with hydrophilic spacers, respectively, after 60 min.	Grafting of dipyridamole to provide a hydrophilic surface that is more hemocompatible.	[28]
Heparin- and phosphorylcholine-grafted PEI/PU membranes	Static: microscopic platelet counting assays	48% reduction of platelet adhesion on PEI-PU membranes. 1000-fold decreases in platelet adhesion for both heparin-tethered and PC-grafted PU membranes.	Tethered PU membranes become more hydrophilic to reduce platelet and protein deposition.	[29]

## Abbreviations:

WHO: World Health Organization
PARs: Protease-Activated Receptors
GPCRs: G-Protein-Coupled Receptors
PU-PEG: Polyethylene Glycol-Polyurethane
ELISA: Enzyme-Linked Immunosorbent Assays
